# Case report: A rare case of anti-PD-1 sintilimab-induced agranulocytosis/severe neutropenia in non-small cell lung cancer and literature review

**DOI:** 10.3389/fonc.2024.1415748

**Published:** 2024-06-18

**Authors:** Yanzhu Qin, Shuaiji Lu, Jingwen Chen, Jing Peng, Jijun Yang

**Affiliations:** ^1^ Department of Pulmonary and Critical Care Medicine-Section 5, Guangzhou Institute of Respiratory Health, The First Affiliated Hospital of Guangzhou Medical University, Guangzhou, Guangdong, China; ^2^ Intensive Care Medicine, Affiliated Loudi Hospital, Hengyang Medical School, University of South China, Loudi, Hunan, China

**Keywords:** immune checkpoint inhibitors, PD-1-immune related adverse effects, sintilimab, agranulocytosis, neutropenia, non-small cell lung cancer

## Abstract

Immune checkpoint inhibitors (ICIs) demonstrate unique advantages in the treatment of lung cancer and are widely used in the era of immunotherapy. However, ICIs can cause adverse reactions. Hematological toxicities induced by immunotherapy are relatively rare. Agranulocytosis, a rare hematologic adverse event associated with immune checkpoint inhibitors, has received limited attention in terms of treatment and patient demographics. Herein, we report the case of a 68-year-old male with non-small cell lung cancer(NSCLC) who received two cycles of programmed cell death-1 (PD-1) antibody sintilimab immunotherapy combined with albumin-bound paclitaxel and carboplatin chemotherapy and one cycle of sintilimab monotherapy. He was diagnosed with grade 4 neutropenia and sepsis (with symptoms of fever and chills) after the first two cycles of treatment. Teicoplanin was promptly initiated as antimicrobial therapy. The patient presented with sudden high fever and developed agranulocytosis on the day of the third cycle of treatment initiation, characterized by an absolute neutrophil count of 0.0×10^9^/L. The patient was treated with granulocyte colony-stimulating factor but did not show improvement. He was then treated with corticosteroids, and absolute neutrophil counts gradually returned to normal levels. To the best of our knowledge, this is the first reported case of sintilimab-induced agranulocytosis in a patient with NSCLC. Sintilimab-induced severe neutropenia or agranulocytosis is a rare side effect that should be distinguished from chemotherapy-induced neutropenia and treated promptly with appropriate therapies; otherwise, the condition may worsen.

## 1 Introduction

In recent years, significant advancements have been made in cancer immunotherapy, particularly with the advent of widely used immune checkpoint inhibitors (ICIs), such as programmed cell death protein 1 (PD-1)/programmed death-ligand 1 (PD-L1) inhibitors and cytotoxic T-lymphocyte-associated antigen 4 (CTLA-4) inhibitors, which can prolong patient survival (1). PD-1 and PD-L1 are inhibitory co-stimulatory molecules that serve as negative immune regulatory factors, playing a pivotal role in adaptive cellular immunity. By selectively binding to the receptor molecule PD-1 on T cells, tumor-expressed PD-L1 is involved in modulating T cell activation and differentiation while also impeding the anti-tumor immune response mediated by T cells (1). Blocking the PD-1/PD-L1 signaling pathway with drugs or monoclonal antibodies has emerged as a novel cancer immunotherapy strategy, demonstrating efficacy in treating various types of cancers, including malignant melanoma, non-small cell lung cancer, renal cell carcinoma, squamous cell carcinoma and gastric cancer (1, 2). Despite the effectiveness of these therapies, the potential for immune-related adverse events (irAEs) cannot be ignored. The main irAEs associated with ICIs include skin, gastrointestinal, pulmonary, hepatic, and endocrine toxicities. Haematological immune-related adverse events (hem-irAEs), including pancytopenia and hemophagocytic lymphohistiocytosis, have rarely been reported. These irAEs affect the process and efficacy of immunotherapy and some can be fatal. A meta-analysis of 9,324 patients showed that 0.94% of patients treated with ICIs experienced neutropenia (3). Sintilimab, a monoclonal antibody against the PD-1 receptor, is increasingly used in patients with previously treated advanced non-small cell lung cancer (NSCLC). To date, hem-irAEs have not been extensively characterized, and there are no reports of neutropenia caused by sintilimab administration nor, standardized treatment and care protocols. Therefore, it is important for healthcare staff to be aware of these fatal irAEs and develop useful strategies to treat them. Here, we report a rare case of severe neutropenia/agranulocytosis after receiving immunotherapy plus chemotherapy and describe the process of differentiation between immune-related and chemotherapy-related neutropenia.

## 2 Case presentation

**Figure 1:**
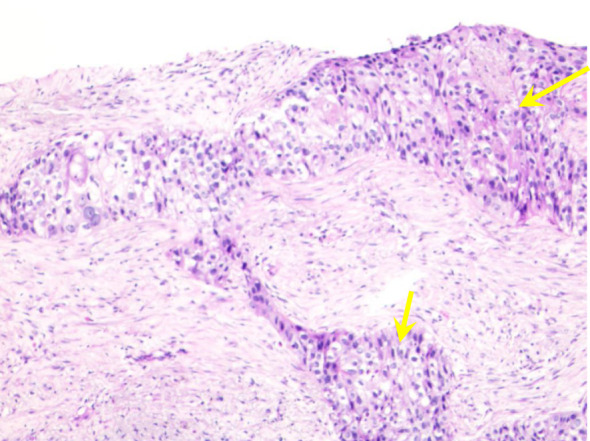
Histopathological characteristics of percutaneous lung biopsy. Microscopic examination reveals irregular nest-like structures of cancer cells with round nuclei, visible small nucleoli, abundant translucent and red-stained cytoplasm, accompanied by areas of necrosis (yellow arrows).

A 68-year-old man was referred to our hospital complaining of a recurrent fever for a month and a 3-day-long chest pain on October 5, 2021. He was previously diagnosed with right lung squamous cell carcinoma (cT3N3M0, stage IIIC) and had a history of deep venous thrombosis for the past five years without previous related treatment. Histopathological analysis suggested non-keratinizing squamous cell carcinoma, with immunohistochemical staining showing EMA(+), CK5/6(+), P40(+), P63(+), TTF-1(-), CK7(-), Napsin A(-), and Ki67(+, 40%) (**Figure 1**). PD-L1 expression in the tumor was negative. The patient had been a smoker for 40 years.

Treatment with sintilimab (200 mg) plus paclitaxel liposomes (240 mg) and carboplatin (0.45 g) was initiated on August 27, 2021, at a local hospital. Five days after the first cycle of drug infusion, the patient reported recurrent fever with peak temperature of 40.5°C, and a blood test revealed grade 3 neutropenia with a white blood cell count(WBC) of 1.7×10^9^/L and an absolute neutrophil count(ANC) of 0.64×10^9^/L. Despite receiving granulocyte-colony stimulating factor (G-CSF) and cephalosporin, the patient experienced no significant improvement in symptoms. Peripheral blood culture results revealed infection with human herpesvirus 5 (HHV-5), Staphylococcus aureus, and Corynebacterium equi, which were considered indicative of sepsis. After the administration of teicoplanin, the fever subsided, and his WBC and ANC returned to normal values (5.04×10^9^/L and 3.32×10^9^/L, respectively).

Subsequently, the patient was discharged after receiving a second dose of sintilimab (200 mg) and oral anticoagulant therapy (edoxaban tosilate tablets, 30mg/day) on September 25, 2021. The following day, he presented with recurrent fever with peak temperature of 40°C. The patient did not return to the hospital until September 30, at which time his WBC and ANC were normal, but elevated inflammatory markers, including C-reactive protein (42.2 mg/L) and Procalcitonin (12.27 ng/mL), indicated sepsis. However, antibiotic therapy proved to be ineffective.

**Figure 2:**
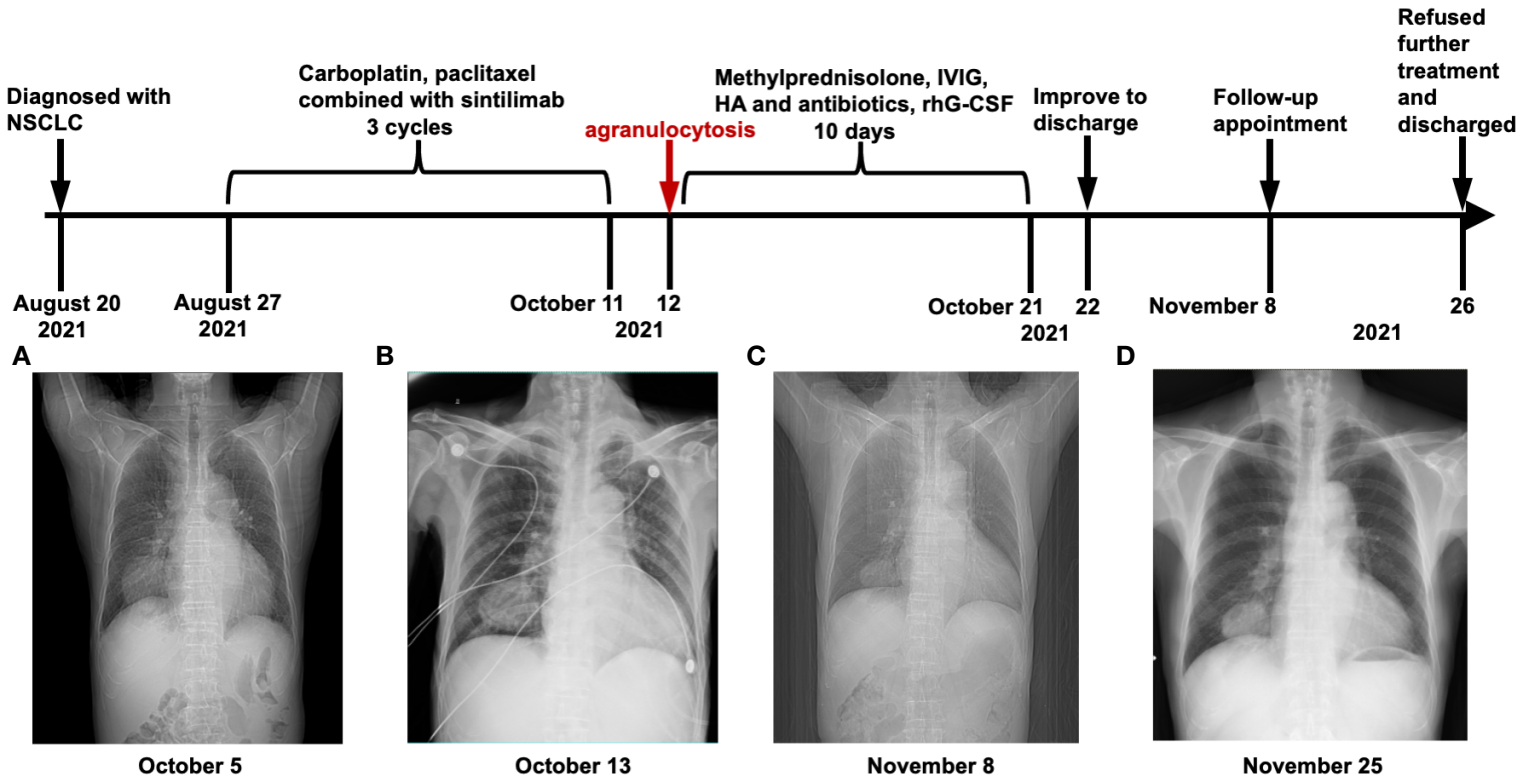
Timeline and CT/DR manifestation. **(A)** Chest imaging at initial hospitalization. **(B)** On the first day after the 3rd cycle of immunotherapy, a chest DR image revealed a potential new growth in the lower right lung with mild surrounding obstructive inflammation. **(C)** In the routine tumor follow-up PET-CT image, a right lower lung posterior basal segment cancer mass volume was similar to the 5 October CT image **(A)**, but with reduced obstructive inflammation. **(D)** Chest DR image upon return for treatment: Reduced right lower lung mass and decreased surrounding inflammation compared to the 13 October DR image **(B)**. (NSCLC, non-small cell lung cancer; HA, human albumin; CT, computed tomography; DR, digital radiography; PET-CT, positron emission tomography-computed tomography; Hb, hemoglobin; rhG-CSF, recombinant human granulocyte colony stimulating factor; IVIG, intravenous immunoglobulin).

**Figure 3:**
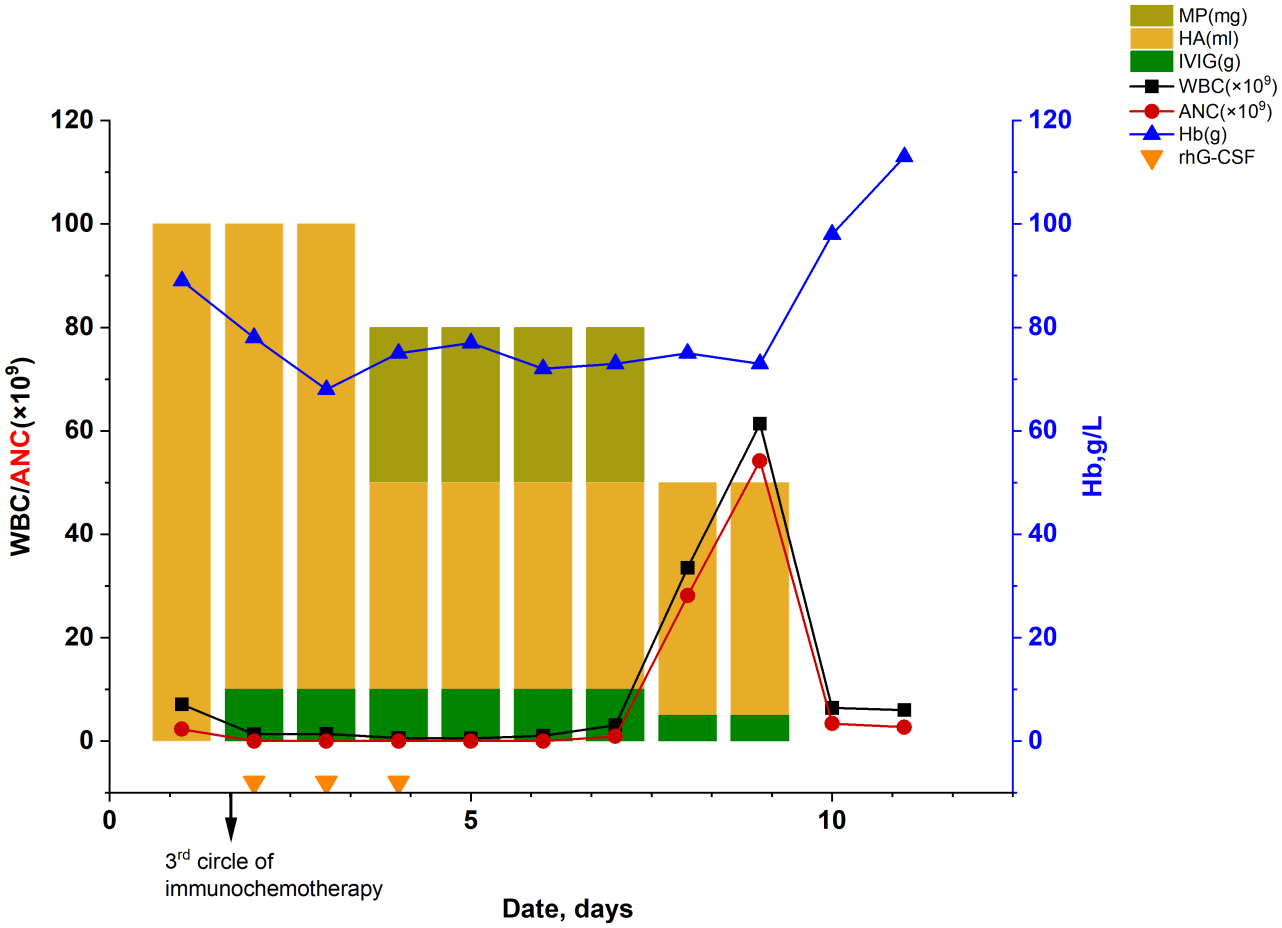
Dynamic changes in the routine blood test. (HA, human albumin; WBC, white blood cell count; ANC, absolute neutrophil count;MP, methylprednisolone; IVIG, intravenous immunoglobulin; Hb, hemoglobin; rhG-CSF, recombinant human granulocyte colony stimulating factor).

The patient suddenly developed chest tightness and pain and was subsequently transferred to our hospital for further treatment. On admission, the patient’s vital signs were within the normal range except for decreased breath sounds in the left lung during physical examination. In addition, the levels of tumor markers were as follows: carcinoembryonic antigen (CEA) at 4.49 ng/mL, cytokeratin 19 fragment (CYFRA 21–1) at 7.22 ng/mL (normal range: 0–3.3 ng/mL), neuron-specific enolase (NSE) at 15.86 ng/mL, and carbohydrate antigen 125(CA125) at 12.41U/mL, carbohydrate antigen 153(CA153) at 31.40 U/mL(normal range: 0–25 U/mL). Notably, both CA153 and CYFRA21–1 were elevated to levels above their respective normal ranges. Re-evaluation of these tumor markers after treatment can serve as a basis for assessing the efficacy of therapy. The inflammatory marker results upon re-evaluation were as follows: C-reactive protein(7.92 mg/L) and Procalcitonin(0.17 ng/mL). A repeat chest CT scan conducted on October 5 revealed a mass measuring approximately 6.1×5.2cm in the right lower lung posterior basal segment (**Figure 2A**), raising concerns of a new neoplasm, with mild perilesional inflammation evident. After 6 days of anticoagulation and heart rate stabilization, coronary artery disease was excluded in this patient using by coronary angiography. Consequently, the patient was subject to the third treatment cycle consisting of carboplatin (0.45g), paclitaxel liposome (270 mg), bevacizumab (300 mg), and sintilimab (200 mg). Soon after midnight he developed a fever with a peak temperature of 40.2°C and exhibited a WBC level of 1.3×10^9^/L with an ANC of 0×10^9^/L indicating agranulocytosis (**Figure 3**). These symptoms were originally considered adverse effects of chemotherapy; hence, the treatment was discontinued immediately. However, his WBC showed progressive decline, reaching a nadir of 0.5×10^9^/L on October 16, with the ANC remaining at 0×10^9^/L throughout; hemoglobin levels were at a low level from October 13 to 21 (**Figure 3**, **Table 1**). Additionally, Digital Radiography (DR) findings on October 13 were consistent with the earlier CT results (**Figure 2B**). This prompted the consideration of the potential bone marrow suppression attributable to immunotherapy. To minimize the risk of infection, the patient was admitted for protective isolation and accommodated in laminar flow beds. Blood cultures were obtained, and the patient was administered intravenous antibiotics (imipenem and cilastatin sodium 1g; vancomycin hydrochloride 500,000 units; caspofungin acetate 50 mg; piperacillin sodium and tazobactam sodium 4.5 g), G-CSF, human albumin(20%, 50 ml), immunoglobulins(5%, reduced from 10 g to 5 g), and blood transfusion(red blood cell suspension 2u). Eating utensils were sterilized and a sodium bicarbonate mouth rinse (sodium bicarbonate and sodium chloride, 250ml, respectively) was given to suppress intraoral disorders. Throughout this period, we closely monitored the patient’s blood parameters using peripheral venous blood analysis. The patient refused to undergo a bone marrow examination; hence, the bone marrow morphology test results were lacking. To explore alternative diagnostic avenues, we conducted various tests, including liver function tests and bacterial cultures, and assessment of drug toxicity. Detection of autoimmune disease revealed that the anti-nuclear antibody (ANA) and fungal galactomannan (GM) tests were negative. Additionally, other diagnostic tests such as the G test, Cytomegalovirus (CMV) DNA, and renal profile were within normal limits. A sputum culture conducted on October 18 was positive for Pseudomonas aeruginosa. Notably, the patient had no prior history of agranulocytosis, and his ANC consistently remained within the normal range before and during cancer treatment. Upon review, the patient exhibited bone marrow suppression within 24 hours of chemotherapy, immunotherapy, and anti-angiogenic therapy, which deviated from the typical peak occurrence of chemotherapy-induced bone marrow suppression. A multidisciplinary team (MDT) meeting was organized, and sintilimab-induced agranulocytosis was diagnosed after excluding evidence of autoimmune disease or tumor invasion of the bone marrow.

**Table 1:** Timeline of blood counts during the third cycle of treatment.

Date	Days of third ICI treatment	WBC(x10^9^/L)	ANC(x10^9^/L)	Hb(g/L)	RBC(x10^12^/L)	PLT(x10^9^/L)
2021–10-6	-5	5.5	3.1	82	3.81	126
2021–10-9	-2	7.1	2.3	89	4.14	261
2021–10-11	0(3^rd^)	–	–	–	–	–
2021–10-13	3	1.3	0	78	3.70	302
2021–10-14	4	1.4	0	68	3.17	225
2021–10-15	5	0.6	0	75	3.44	203
2021–10-16	6	0.5	0	77	3.45	188
2021–10-17	7	1	0	72	3.22	165
2021–10-18	8	3.1	0.9	73	3.37	147
2021–10-19	9	33.5	28.1	75	3.43	136
2021–10-21	11	61.4	54.2	73	3.47	113
Normal Ranges	–	4.0–10.0	1.0–8.0	120–160	4.5–5.5	100–400

WBC, white blood cells; ANC, absolute neutrophil count; Hb, hemoglobin; RBC, red blood cells; PLT, platelets; -, no data is available.

Considering the patient’s history of neutropenia induced by platinum-based chemotherapy, we initiated long-term prophylactic use of recombinant human G-CSF (rhG-CSF) until the ANC returned to normal or near-normal laboratory reference values from its nadir. The patient was started on a treatment regimen consisting of rhG-CSF(5μg/kg, hypodermic injection) for a period of 9 days and methylprednisolone at a daily dosage of 80 mg for 3 days, followed by a subsequent dose reduction to 40 mg over the course of 2 days. Approximately 10 days after the third administration of combined sintilimab therapy, the neutrophil count returned to normal, and no fever was observed (**Figure 3**, it is noted that no statistical analysis was employed for the comparison between the lines). The patient was discharged on October 22 and scheduled for a follow-up appointment.

The best response to the third combination of immunotherapies was stable disease(SD). Consequently, the rapid decline in ANC was suspected to be induced by anti-PD-1 antibody. Follow-up positron emission tomography CT (PET-CT) on November 8 revealed that the tumor volume in the right lower lobe peripheral basal segment remained largely unchanged compared to the previous assessment(**Figure 2C**). Additionally, there was a slight reduction in distal obstructive pneumonia compared to the prior examination.

When the patient returned to our hospital to receive the fourth cycle of chemotherapy on November 25, he no longer received treatment for the previous immune-related responses. Chest CT revealed a decreased mass, measuring approximately 5.6×4.8 cm in the right lower lung, in comparison to the previous assessment, with a slight reduction in perilesional obstructive inflammation (**Figure 2D**). Subsequent testing of tumor markers revealed carcinoembryonic antigen (CEA) at 4.63 ng/mL, cytokeratin 19 fragment (CYFRA 21–1) at 4.45 ng/mL (normal range: 0–3.3 ng/mL), neuron-specific enolase (NSE) at 14.89 ng/mL, carbohydrate antigen 125(CA125) at 11.9U/mL, and carbohydrate antigen 153(CA153) at 21.10 U/mL. Compared to pre-treatment results, all tumor markers decreased, with CA153 falling within the normal range; CYFRA21–1 also decreased but remained above normal, indicating, along with the CT results, that the patient showed some response to the treatment; however, the tumor remained active to some extent. The patient experienced fever (38.3°C) again and general malaise following the administration of paclitaxel liposome (270 mg). These symptoms alleviated after discontinuation of the medication. The patient’s white blood cell count was 6.0×10^9^/L, and ANC was 2.7×10^9^/L. Ultimately, the patient chose to discontinue the treatment and was discharged on November 26. During a follow-up telephone consultation, the patient continued to receive regular antineoplastic treatment at the local hospital every three weeks but did not opt for immunotherapy rechallenge.

## 3 Discussion

NSCLC is the most common clinical subtype of lung cancer, accounting for up to 85% of all lung cancer cases, and over 30% of patients with lung cancer are diagnosed at a locally advanced stage (4). Moreover, conventional clinical treatments often yield suboptimal efficacy in this subset of lung cancer patients, leading to a poor prognosis. Immune checkpoint inhibitors have been approved for the treatment of various malignancies, including lung cancer. Sintilimab can effectively bind to PD-1 and interfere with the interaction between PD-1 and its ligand PD-L1, thereby activating T cell function and exerting antitumor effects. Clinical studies have demonstrated favorable therapeutic efficacy in patients with advanced NSCLC (5). However, the activation of the immune system can contribute to toxic reactions in multiple effector organs, thus affecting organs such as the endocrine system and gastrointestinal tract. Neutropenia, a common adverse reaction to chemotherapy treatment, can easily be confused with ICI-induced agranulocytosis when chemotherapy is used in combination with immunotherapy. This confusion may result in the misuse of treatment and pose a threat to the patient’s health and well-being.

The diagnostic criteria and mechanisms underlying ICI-associated neutropenia remain unclear. Currently, the most efficient diagnostic approach for investigating neutropenia typically involves a bone marrow examination. However, in certain cases where the etiology can be explained by patient history and basic laboratory panels, bone marrow examination may not always be clinically necessary for elderly patients. The critical objective was to rule out other potential causes of neutropenia, confirm the diagnosis, and evaluate its severity. In our patient, who had no prior history of rheumatic autoimmune diseases and tested negative for anti-nuclear antibodies upon admission, the development of neutropenia following the first and third cycle of chemotherapy in combination with sintilimab treatment raised concerns. Although the patient exhibited severe decreased WBC and ANC, moderate reduction in red blood cells, and normal platelet count (**Table 1**), he presented with symptoms of fever and fatigue without bone pain. These findings suggest the possibility of bone marrow infiltration but are not conclusive. Furthermore, the restoration of ANC following G-CSF and antibiotics administration strongly supports the diagnosis of drug-induced agranulocytosis rather than bone marrow infiltration. It is worth noting that the use of medication in our 68-year-old patient carries inherent risks for neutropenia, especially given the increased susceptibility of elderly individuals to chemotherapy-induced neutropenia (6). Although paclitaxel liposomes and carboplatin have been previously associated with neutropenia (7, 8), it is noteworthy that the patient experienced fever even after discontinuing sintilimab during forth cycle of treatment, which may be attributed to prior exposure to platinum-based chemotherapy, advanced or metastatic disease stage, previous chemotherapy exposure, or immune-related effects stemming from immunotherapy.

In this particular case, agranulocytosis was detected within 24 h of the third combined ICI treatment, suggesting that immunotherapy may have increased the risk of myelosuppression. The Chinese Society of Clinical Oncology (CSCO) guidelines for the standardized management of tumor chemoradiotherapy-related neutropenia show that ANC changes in chemotherapy-related neutropenia follow a U-shaped trend approximately 7–14 days after chemotherapy. These levels generally return to normal within 14–21 days (9, 10). In contrast, ICI-related neutropenia can manifest at any time (11) and often presents as grade 3 or 4 neutropenia, which can normalize within two weeks with the use of G-CSF and methylprednisolone (12). The high percentage of patients treated with combined ICI (70%) and earlier and more frequent laboratory testing in these patients indicate that immune-related adverse events generally occur earlier in patients receiving combined ICI (13). Moreover, following administration of the second dose of sintilimab monotherapy, the patient exhibited pyrexia and elevated levels of inflammatory markers on the subsequent day, potentially suggestive of transient neutropenia. Therefore, it is reasonable to hypothesize that the observed neutropenia in this patient following two cycles of chemo-sintilimab combination therapy was likely induced by the synergistic effects of chemotherapeutic agents and ICI. Sintilimab appears to have played a predominant role in precipitating agranulocytosis during the third treatment cycle.

Hem-irAEs can lead to severe neutropenia in patients receiving combined ICIs, rendering them susceptible to bacterial and fungal infections (14). These infections can escalate to septicemia and increase the risk of mortality (15). Notably, four reported cases have been associated with severe neutropenia related to anti-PD-1 antibodies in patients with advanced NSCLC, including three cases linked to nivolumab (16–18) and one to atezolizumab (19). To our knowledge, no case reports of hematotoxicity induced by a combination of sintilimab, carboplatin, and paclitaxel have been published in PubMed, and the pathological features of this combination therapy are not clear. The mechanisms underlying immune-related adverse events induced by PD-1/PD-L1 inhibitors remain poorly understood. Similar to other immune-related adverse events, hematological toxicity is believed to involve the generation of autoreactive T and B cells along with a decrease in the regulatory T cell phenotype (3). Furthermore, the fourth episode of fever could potentially be attributed to acute hypersensitivity reactions to paclitaxel, which commonly manifests immediately after drug administration. These reactions are associated with the release of proinflammatory cytokines, including IL-6 and TNF-α, which are collectively known as cytokine storms.

The management of irAEs typically involves systemic steroids and symptomatic therapies. Corticosteroids possess immunosuppressive characteristics by exerting pleiotropic effects on the activation, differentiation, and movement of T cells. They inhibit the IL-2 induced activation of effector T cells while promoting the expansion of regulatory T-cells (20). When determining whether to discontinue therapy and administer steroids based on the severity of hem-irAEs (21), consideration should also be given to the potential impact on other irAEs. In our study, the patient with grade 4 neutropenia initially received blood transfusion, antibiotics, and G-CSF therapy. However, methylprednisolone was subsequently administered after failure of initial therapy. This decision was primarily driven by uncertainty in the diagnosis of hem-irAEs, resulting in delayed initiation of steroid therapy. The patient’s response to treatment further substantiates the occurrence of immunotherapy-induced neutropenia, given that while chemotherapy induced neutropenia usually improves with the use of antibiotics and G-CSF, immunotherapy-induced neutropenia tends to resolve after steroid administration. G-CSF-based agents can promote the release of mature neutrophils from marginal pools into the peripheral blood and accelerate the differentiation of committed neutrophil precursors in the bone marrow (22). While certain irAEs do not necessarily necessitate the discontinuation of ICI therapy (23), hem-irAEs appear to persist even in the presence of ongoing ICI therapy. In the present case, the patient experienced fever after each ICI treatment, ultimately leading to the patient’s decision to discontinue long-term treatment, which in turn accelerated disease progression. Some researchers suggest that downregulation of the immune system with systemic steroids is not recommended for use in immune-related neutropenia (11, 15), whereas others recommend their use with caution (14, 24–26) and in the absence of any evidence of infection (25). It should be noted that steroid use can increase susceptibility to secondary infections.

In conclusion, this case highlights the occurrence of neutropenia, a hematological toxicity, induced by ICIs in combination with chemotherapy. Importantly, the patient responded successfully to short-term steroid therapy. Although severe neutropenia is rare, it is a critical and potentially life-threatening condition requiring prompt clinical intervention.

## Data availability statement

The raw data supporting the conclusions of this article will be made available by the authors, without undue reservation.

## Ethics statement

The studies involving humans were approved by The First Affiliated Hospital of Guangzhou Medical University Ethics Committee. The studies were conducted in accordance with the local legislation and institutional requirements. The participants provided their written informed consent to participate in this study. Written informed consent was obtained from the individual(s) for the publication of any potentially identifiable images or data included in this article.

## Author contributions

YQ: Conceptualization, Writing – original draft. SL: Data curation, Investigation, Writing – original draft. JC: Investigation, Methodology, Writing – original draft. JP: Resources, Writing – review & editing. JY: Project administration, Supervision, Writing – review & editing.
